# A life course approach to reproductive health: Theory and methods

**DOI:** 10.1016/j.maturitas.2009.12.009

**Published:** 2010-02

**Authors:** Gita D. Mishra, Rachel Cooper, Diana Kuh

**Affiliations:** MRC Unit for Lifelong Health and Ageing, Department of Epidemiology and Public Health, University College and Royal Free Medical School, 33 Bedford Place, London WC1B 5JU, United Kingdom

**Keywords:** Life course models, Women's health, Critical periods, Sensitive periods

## Abstract

Taking a life course approach to the study of reproductive health involves the investigation of factors across life and, also across generations, that influence the timing of menarche, fertility, pregnancy outcomes, gynaecological disorders, and age at menopause. It also recognises the important influence of reproductive health on chronic disease risk in later life. Published literature supports the use of an integrated life course approach to study reproductive health, which examines the whole life course, considers the continuity of reproductive health and the interrelationship between the different markers of this. This is in contrast to more traditional approaches that tend to focus only on contemporary risk factors and which consider each marker of reproductive health separately. For instance, we found evidence linking early life factors such as growth, socioeconomic conditions, and parental divorce with ages at menarche and menopause, although the nature of the relationship differs. We discuss the different theoretical models that are used within life course epidemiology and which postulate pathways linking exposures across the life course to health outcomes, using examples of relevance to the study of reproductive health. These highlight the importance of examining timing of exposures, such as during critical periods in early life, and the temporal order of exposures. How life course frameworks of reproductive health can be developed to help identify hypotheses to be tested is also demonstrated. This approach has implications for the development of effective health policy that moves beyond identifying not only the type of intervention but also the most appropriate time across life to intervene.

## 1 Introduction

Menarche heralds the beginning of a female's reproductive life with menopause signalling its end. The timing of these two events is often used to provide an indication of a woman's reproductive health. Other events, such as childbirth and gynaecological surgery, and characteristics, such as menstrual regularity, gynaecological problems, pregnancy complications and offspring birthweight, are used to characterise reproductive health further [1]. Each of the different components of a woman's reproductive health can be, and often are, considered separately. Many, however, are strongly associated with each other, as they act as indicators of the same underlying traits, for example endogenous hormone levels or subfertility, or, are on the same causal pathways.

In studying reproductive health it is first necessary to identify which indicators to use and consider how they may be related to each other. If the aim is then to investigate factors which may influence these, it is important to consider that factors across life, from conception onwards, have been shown to be associated with reproductive health outcomes [1,2]. This is exemplified in the findings from a recent review of the literature on the early life predictors of age at menarche and menopause [2]. This found a range of factors in early life to be associated with the timing of menarche and menopause. For example, the main factors in early life found to be associated with early menarche were: faster growth rates during childhood [3,4]; higher childhood socioeconomic position [5–9]; family conflict and parental divorce [10–12]; presence of a stepfather [13] and; exposure to stressors such as war shortly before menarche [14,15]. This review also showed that while the relationships between many adult environmental factors and timing of menopause have been investigated, only cigarette smoking and nulliparity have consistently been related to an earlier menopause [16–21]. However, some studies have demonstrated a link between early life factors and early menopause; these included not having been breastfed, poor early growth, poor socioeconomic conditions, lower childhood cognitive abilities, and parental divorce in childhood [22,23].

In addition to evidence of associations between factors across life, from early life onwards, and reproductive health outcomes, there is also growing evidence that a woman's reproductive health is linked to the reproductive characteristics of previous generations [24]. Taking timing of menarche and menopause as examples again, there is evidence for a positive correlation between mother's and daughter's age at menarche [24] and family and twin studies have indicated that the genetic effect on timing of menopause is considerable, with estimates of heritability ranging from 30% to 85% [25,26]. This is supported by evidence from cross-sectional and cohort studies that a woman's age at menopause is strongly associated with her mother's reported age at menopause [19,23,25–29]. By taking all of this evidence into consideration we highlight the importance of a life course perspective. Of note however is that while the field of life course epidemiology is considered to be relatively new, having been coined as a term in the 1990s, there has been a longstanding interest in the long-term effects of early life exposures on adult disease risk [30].

Further justification for the use of a life course perspective comes from the increasing recognition that, as well as being integral to her overall health and wellbeing, a woman's reproductive health is a sentinel of chronic disease in later life [1,31–33]. It has long been acknowledged that earlier menarche, later menopause and other reproductive characteristics, for example nulliparity, are associated with increased risk of some cancers, including breast [34,35] and endometrial, [36–38] and lower risk of osteoporosis. Furthermore, it has been shown that markers of reproductive health, such as pregnancy complications including pre-eclampsia, pregnancy-induced hypertension, gestational diabetes, preterm delivery, and having a low birthweight baby, indicate future risk of cardiovascular disease [1,31,39].

In order to apply a life course perspective appropriately there is a need to understand the theories and models that underpin this approach.

## 2 A life course approach to reproductive health

A life course approach examines how biological (including genetics), behavioural and social factors throughout life, and across generations [40], act independently, cumulatively and interactively to influence health. A life course approach to reproductive health asks a range of questions that are relevant to the development of health policy. For example, does birthweight influence the age of menarche and of menopause? Does maternal stress during pregnancy influence the development of polycystic ovary syndrome in female offspring? What is the influence of childhood growth on age at menopause and is this modified by adult body size? Could the link between reproductive health and other chronic diseases be due to a common set of factors that affects them both and, if so, when and what is the best way to intervene? What is the impact of grandmother's fertility rate on that of the granddaughter's? Would preventing maternal gestational diabetes provide the most cost-effective means of reducing the risk of gestational diabetes in the offspring?

At the heart of this life course perspective lies a theoretical framework that assumes and tests for a temporal ordering of exposure variables and their inter-relationships with the outcome measure, both directly and through intermediary (mediating or modifying) variables [40,41].

## 3 Life course epidemiology: theoretical models (with relevant examples)

**Fig. 1:**
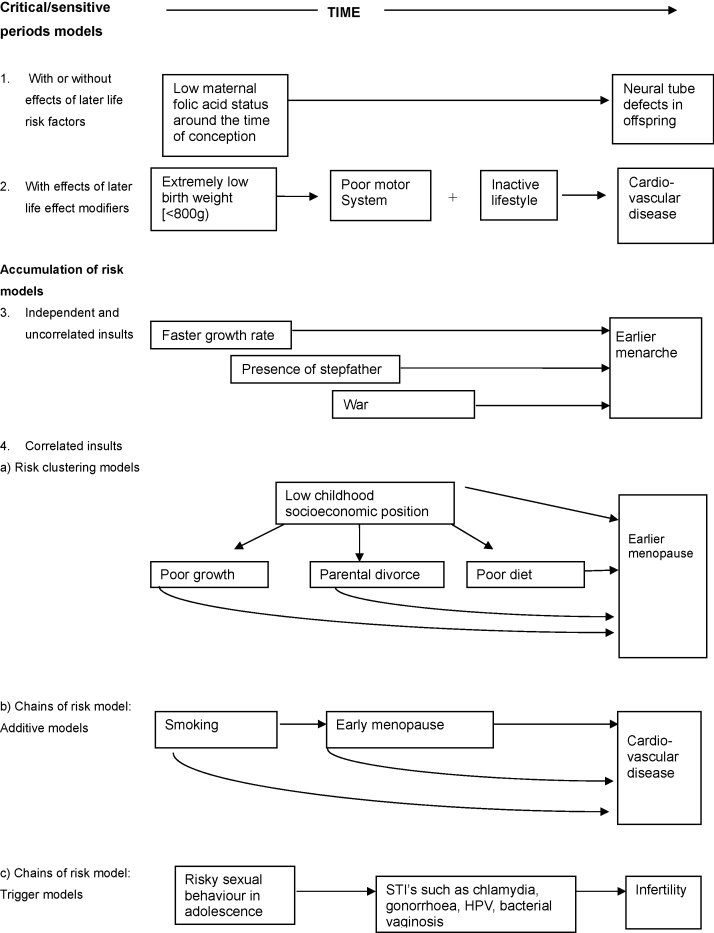
Life course models with illustrative examples.

The underlying purpose of life course epidemiology is to build and test theoretical models that postulate pathways linking exposures across the life course to health outcomes [41]. Given the wide range of exposures and the potential importance of their timing and duration, exposures may be acting to influence disease risk on a variety of different pathways. Four broad hypothetical life course models that can operate for exposures acting at different points across the life course have been proposed (Fig. 1) [40], these are the: critical period model; the critical period model with later life effect modifiers; accumulation of risk model; and chains of risk model each of which is described in more detail below.

The critical period model pays attention to the timing of an exposure and assumes that the irreversible changes in body systems that occur during a particularly vulnerable phase of life, usually during early development, have implications for later health [30,41]. The basic critical period model, also known as biological programming or as a latency model, underlies the fetal origins of adult disease hypothesis [41]. An example of this, of relevance to the study of reproductive health, comes from the well-known randomized double-blind prevention trial that showed intake of folic acid supplementation around the time of conception, but not later in pregnanacy, can prevent neural tube defects in the offspring [42] (Fig. 1, model 1).

An expanded version of the critical period model includes the possibility of exposures in early life interacting with later life exposures, thereby either enhancing or decreasing the risk of chronic disease in later life; this model can be described as the critical period with later effect modifiers [40,41]. For instance, the risk of cardiovascular disease in midlife of those with low birth weight (<800 g) could be due to the interactive effects of low birth weight on the motor system together with a more inactive lifestyle (Fig. 1, model 2) [43,44].

In contrast to the critical period models, the accumulation of risk model assumes that cumulative insults or exposures during the life course increase the risk of health later in life irrespective of their timing. This idea is similar to the notion of allostatic load [41] so that as the number, duration, and severity of exposures increase, there is cumulative damage to biological systems. Risk exposures may cause long-term, gradual damage to health in separate and independent ways (accumulation model with independent and uncorrelated insults). For example, an individual may experience a variety of unrelated exposures, such as having a higher growth rate, the presence of a stepfather, and being exposed to war, all of which cumulatively impact on age at menarche independently of each other (Fig. 1, model 3) [2].

While it is plausible that a range of different exposures will act independently of each other and accumulate to influence disease risk, it is more common for them to cluster together in socially patterned ways. The accumulation model with risk clustering takes this into account. For example, low childhood socioeconomic position is associated with poorer growth, more family stress and inadequate diet all of which may increase risk of earlier menopause (Fig. 1, model 4a) [30]. Here, understanding the effects of childhood socioeconomic position by identifying the specific aspects of the early physical and psychosocial environments or possible mechanisms (such as nutrition, infection or stress) that are associated with age at menopause may provide further etiological insights.

The chain of risk model is a special version of the accumulation model and refers to a sequence of linked events where one adverse (or beneficial) exposure or experience tends to lead to another, and so on. For example, smoking may lead to early menopause, which in turn may increase the likelihood of developing heart disease. Here, each exposure in a chain of risk may not only increase the risk of subsequent exposure in a probabilistic way, but may also have an independent additive effect on later health (Fig. 1, model 4b). Alternatively, it may be that only the final link in the chain leads to the adverse outcome, for example risky sexual behaviour is associated with increased risk of sexually transmitted infections which are associated with infertility [45] (trigger effect) (Fig. 1, model 4c).

### 3.1 Critical period versus sensitive periods

As discussed above, in life course epidemiology, different stages of life and development are referred to as critical or sensitive periods. Making the distinction between these is important but often difficult to do. To elaborate, a critical period is defined as a limited time window in which an exposure can have adverse or protective effects on development and subsequent disease outcomes [41]. Outside this window, there is no excess disease risk associated with the exposure.

A sensitive period is a time period when an exposure has a stronger effect on development and hence disease risk than it would at other times. For instance, in a recent review it was found that when compared with nonsmokers, current smokers had a greater reduction in age at menopause than former smokers [46]. This suggests that perimenopause is a *sensitive period* when the effect of smoking may be more important than smoking history in explaining an earlier onset of menopause.

Critical periods may be more evident for chronic disease risk associated with developmental mechanisms in biological subsystems, whereas sensitive periods are likely to be more common in behavioural development [41].

## 4 Life course framework to reproductive health

**Fig. 2:**
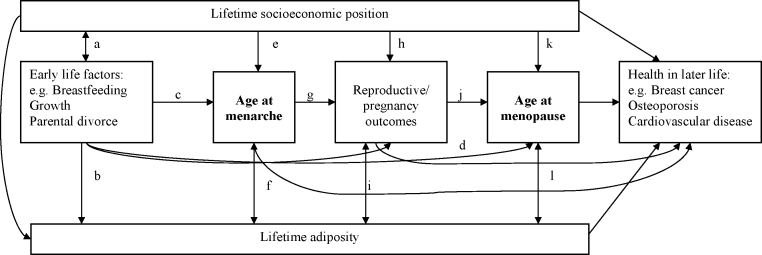
An example of a schematic representation of biological and social factors acting across the life course that may influence the timing of menarche and menopause.

In most cases, once the specific life course associations to be tested using empirical data and the theoretical models most likely to underpin these have been identified it is important to place these in the context of a wider life course framework. In this framework we are able to acknowledge, even if we cannot test all the associations which are depicted, the potential role of factors across life in explaining the specific associations under investigation. For example, Fig. 2 shows the pathways linking biological and social factors across life to reproductive health. Taking age at menopause as our outcome of interest, paths (a), (b) and (d) depict the effect of factors operating earlier in life, while paths (k) and (l) represent factors operating closer to the time of menopause (see Fig. 2). If the associations of early life factors such as breastfeeding, parental divorce and growth with timing of menopause (path (d)) are to be tested, it is useful to consider not only the different pathways on which these factors may operate, but to take into consideration the continuity of reproductive health across life (paths (c), (g), and (j)) and other lifetime factors which may act as confounders or mediators of these associations (paths (a) and (b)) (see Fig. 2). Likewise, if we are to consider the association between timing of menopause and cardiovascular disease risk in later life it is important to make similar considerations, including taking into account the effects of pre-existing cardiovascular risk prior to menopause [47].

## 5 Methodological challenges encountered in studying the life course

In recent years, there have been developments of new statistical approaches and epidemiological thinking in relation to causal models that can be usefully applied to etiological questions framed within a life course paradigm [48–50]. We demonstrated that the critical period models, accumulation models, the effect modification models (such the critical period with later modifier) were each a special case of the saturated model, which contains the effects of all possible combinations of the exposure measures across the life course [50]. By comparing the model fit of a set of nested models – each corresponding to the accumulation, critical period, and effect modification hypotheses – to an all-inclusive (saturated) model, the model that best describes the data can be selected. As the life course models described above are not mutually exclusive and may operate simultaneously, this standard approach to model building can provide further clues to the processes operating across the life course. However, the development of standardised and acceptable methods of combining the different types of cumulative exposures are some of the many methodological challenges that still need addressing.

It remains essential to have an understanding of the biological mechanisms underlying the effect of exposures on specific reproductive health outcomes upon which to base the statistical modeling. Regardless of which statistical methods have been selected – including structural equations models, path analysis, G-estimation, and multi-level models – issues of measurement error, missing data, survival bias, and confounding factors are inevitable in life course studies and hence results may still be biased and caution is required in their interpretation [41].

## 6 Conclusions and future research

In this article, we have highlighted how a life course framework encourages one to consider and test for a temporal ordering of exposure variables across life and their inter-relationships with the outcome measure, both directly and through intermediary variables. We also suggest that the use of a life course approach may provide a better understanding of women's reproductive health. There is evidence that the study of reproductive health would benefit from an integrated approach covering the whole life course, rather than an approach that restricts itself to the study of contemporary risk factors, or which considers each reproductive outcome separately. For instance, we found consistent evidence, though the nature of relationships differ, linking early life factors, such as growth, socioeconomic conditions, and parental divorce with both ages at menarche and menopause.

As the value of the life course approach to health is increasingly recognised, a number of areas have emerged as important directions for future research. Relatively little work has been done, partly due to the lack of prospective data, to study the extent to which childhood nutrition underlies the relationship between growth and age at menarche or menopause. Further work is also required to disentangle the associations between the different types of stressors in early life and pubertal timing. The effects of factors across the life course on markers of other reproductive health outcomes such as polycystic ovary syndrome, fertility and gynaecological disorders also need to be studied. The full impact on reproductive health, of the rise in childhood obesity, and the delay in the age at first birth in the industrialised world is also yet to be realised.

Family-based studies (intergenerational, sibling and twin studies) across the life course can be used to test specific causal mechanisms and life course models as they can help understand whether the timing of risk factors (critical and sensitive periods) are important and establish the role of heritability [51]. By moving beyond associations, to understanding the underlying mechanisms that determine reproductive health and the relationships with chronic disease, we will strengthen our ability to predict population outcomes and make timely interventions that can benefit current and future generations of women.

## Contributors

GDM, RC and DK contributed to the design of the research, read, edited, and approved the final manuscript.

## Conflict of interest

None declared.

## Funding

GM and DK are supported by the Medical Research Council. RC is funded by the New Dynamics of Ageing (RES-353-25-0001).

## Provenance

Commissioned and externally peer reviewed.
